# The Advance and Correlation of *KRAS* Mutation With the Fertility-Preservation Treatment of Endometrial Cancer in the Background of Molecular Classification Application

**DOI:** 10.3389/pore.2021.1609906

**Published:** 2021-12-16

**Authors:** KeXuan Yu, Yiqin Wang

**Affiliations:** Department of Pathology, Obstetrics and Gynecology Hospital of Fudan University, Shanghai, China

**Keywords:** KRAS mutation, endometrial cancer, fertility-preservation, fertility-spared, conservative treatment, molecular classification

## Abstract

The annually increasing incidence of endometrial cancer in younger women has created a growing demand for fertility preservation. However, the diverse therapeutic efficacy among patients under the same histological subtype and the same tumor grade suggests the potential interference of the innate molecular characteristics. The molecular classification has now been applied in clinical practice and might help to stratify the endometrial cancer patients and individualize the therapy, but the candidates for the fertility-spared treatment are most likely to be subdivided in the subgroup lacking the specific signature. *KRAS* mutation has been linked to the malignant transition of the endometrium, while its role in molecular classification and fertility preservation is vague. Here, we mainly review the advance of molecular classification and the role of *KRAS* in endometrial cancer, as well as their correlation with fertility-preservation treatment.

## Introduction

Endometrial cancer (EC) ranks as the second most common malignancy originating from the female reproductive tract, the incidence of which in the population between 20 and 44 years old is increasing globally and accounts for approximately 8.6% of all 417,367 new cases (1). In China, EC is already the most common malignant tumor of the female reproductive tract in developed areas (2), and the proportion of patients with EC diagnosed between 20–44 years old is up to 14.2% (1). The increasing incidence in the younger population thus creates demands of reproductive-aged patients for fertility preservation, while the therapeutic efficacy of conservative treatment varies even in the same histological subtype and the same treatment strategy, suggesting the underlying high molecular heterogeneity of lesions. In 2013, a new molecular classification was proposed and has now been introduced into the clinical-pathological diagnosis in the latest version of NCCN (National Comprehensive Cancer Network) guideline. Patients suitable for the fertility-spared treatment are most likely subdivided into the molecular subtype of which the specific molecular signatures are still under exploration. The Kirsten rat sarcoma viral oncogene homolog (*KRAS*) represents the most prevalent oncogene in human cancers, the mutation of which is reported to predict the carcinogenesis as well as the invasive progression of type I EC (3), suggesting that *KRAS* might be applied as a potential monitoring target for the fertility-preservation treatment of the early stage type I EC. Here we mainly discuss the advance of the molecular classification of EC and the potential link of *KRAS* mutation to the oncologic outcomes of conservative treatment in early EC patients.

## The Conservative Treatment of EC

In 1983, with an understanding of the endocrine and metabolic pathogenesis of EC, Bokham broadly divided EC into two subtypes: the estrogen-dependent (most are histologically diagnosed as endometrioid adenocarcinoma) type I and the estrogen-independent (mainly serous and clear cell carcinoma) type II (4). In 1997, Kim et al. first reported that 13 of 21 (62%) patients who were treated with progestin alone for type I EC had an initial response to progestins (5), beginning the exploration of the criteria for the conservative treatment. At present, relevant guidelines for fertility preservation treatment of EC have been launched, including the NCCN^1^ (6), the Gynecological Oncology Committee of Chinese Anti-Cancer Association (7), and the European Society of Gynecological Oncology (ESGO) (8–11). The main indications agreed by the above guidelines/expert consensus are as follows:

1) Well-differentiated (grade 1) endometrioid adenocarcinoma confirmed by expert pathology review,
2) Disease limited to the endometrium on imaging,
3) Absence of suspicious or metastatic disease on imaging,
4) No contraindications to medical therapy or pregnancy, and
5) Patients should undergo counseling as the fertility-sparing option is not standard of care for the treatment of endometrial carcinoma.

The main treatment strategy is the use of progestin-based oral drugs [megestrol acetate (MA) or medroxyprogesterone acetate (MPA)] or the levonorgestrel (LNG) -intrauterine system/device (IUS/IUD) with or without the hysteroscopic resection of the lesions, accompanied by the evaluation based on the image and the pathological status of the endometrium every 3–6 months. The complete response (CR) is defined as the EC lesions being totally diminished after 6 months of therapy (12) and no response (NR) or failure should be considered when the EC still persists after 6–12 months of therapy and a hysterectomy is recommended.

Although the effectiveness of the fertility-spared treatment has now been widely recognized, the overall CR rate of conservative treatment is reported to fluctuate from 50.0 to 90.0% (13–15). Despite the different use of progestins and hysteroscopy, a center reported that even under the same treatment and histological type, the CR rate was only 40.0% in patients who were diagnosed as grade 1 endometrioid adenocarcinoma with minimal myometrial infiltration (16). These results indicate that the EC cases with similar morphological features show individualized therapeutic efficacy under the same therapy, suggesting that the molecular signature of the tumor might be essential for the implementation and assessment of conservative therapy.

## The Molecular Classification of EC

In 2013, The Cancer Genome Atlas (TCGA) using an integrated multi-genomic transcriptomic and proteomic platform classified EC into four subgroups (17): the POLE subgroup (7%) had characteristical mutations in the exonuclease domain of *POLE* with a unique C > A transition, which was mostly composed of high grade endometrioid tumors and exhibited the best progression-free survival (PFS); the CN-H subgroup (26%) was characterized by frequent (over 90%) somatic mutations in *TP53* with extensive somatic copy number alterations, comprised of 9% of the endometrioid (56% of grade 3 and 44% of grade 1/2 EC) and almost all of the serous (41/42), and exhibited the poorest outcome; the MSI subgroup (28%) was characterized by MSI and frequent *MLH1* promoter hypermethylation and primarily consisted of endometrioid carcinomas, 62% of which were diagnosed as low-grade (grade 1 and grade 2) endometrioid tumors; finally, the CN-L subgroup had the largest proportion (39%) in EC with a relatively stable microsatellite status, more complex molecular characteristics, lower mutation frequency and somatic copy number alterations, and increased progesterone receptor expression. The CN-L subgroup was dominantly enriched for the low-grade endometrioid carcinomas (94%) but showed worse PFS than either the POLE or the MSI subgroup.

The value of molecular classification of EC has been identified by the subsequent studies and is recommended in the newest version of NCCN guidelines and WHO classification. Considering the equipment in most pathology laboratories, the ProMisE strategy proposed using the immunohistochemistry (IHC) of MMR protein as a surrogate to screen the MSI subgroup, as well as the IHC of TP53 to the CN-H subgroup (18–20). The patients under fertility-preservation treatment might also profit from the molecular classification. Since the conventional progesterone receptor and estrogen receptor isoforms showed insufficient predictive accuracy (21), the ProMisE strategy has also been practiced in the population of fertility preservation to identify the MSI status and the potential TP53 abnormality (22–24). *POLE* mutation could be detected only by sequencing (the next-generation sequencing or Sanger sequencing), considering its superior prognostic value, and it might be of use for young patients with strict fertility-spared conditions.

However, the most troubling problem might be the CN-L subgroup, as the patients entitled to the fertility-spared treatment are mainly (99%) aggregated in the subgroups of POLE, MSI, and CN-L, and nearly 62% of them fall into the CN-L subgroup (17). TCGA data showed that the significantly mutated genes in this subgroup included *PTEN*, *PIK3CA*, *PIK3R1*, *ARID1A*, *CTCF*, *CTNNB1*, and *KRAS*. Recently, long non-coding RNAs (lncRNAs) have been reported to be upregulated or downregulated in ECs compared to normal tissues and their dysregulation has been linked to tumor grade, FIGO stage, the depth of myometrial invasion, lymph node metastasis, and patient survival (25). LncRNA homeobox transcript antisense intergenic RNA (HOTAIR) has been confirmed to bind with lysine-specific demethylase 1 to demethylate the histone 3 lysine 4 on progesterone receptor B promotor, thus inhibiting progesterone receptor B transcription and decreasing progesterone sensitivity (26), and long non-coding RNA nuclear enriched abundant transcript 1 (LncRNA-NEAT1) promotes EC proliferation and invasion by regulating the miR-144-3p/EZH2 axis (27).

Complex molecular characteristics of the CN-L subgroup might lead to the high differences in response to the fertility-spared treatment. In a recent study performing molecular analysis on 15 fully evaluable EC patients, three of the eight patients in the CN-L subgroup respectively relapsed at the 8th, 11th, and 41st months of conservative treatment (22). Another study showed similar results (24), suggesting the lack of the effective molecular signature of the CN-L subgroup might impair the value of the molecular strategy to stratify the patients under fertility-preservation treatment. All these problems suggest that further molecular stratification to subdivide the patients is urgently needed.

## KRAS Mutations in EC and its Potential Value in the Fertility-Spared Treatment

*RAS* (*KRAS*, *NRAS*, and *HRAS*) is one of the most frequently mutated gene families in cancers, the protein form of which is a kind of membrane-bound GTPase switching to transmit signals within cells (28), controlling processes like cellular growth, cell proliferation, cell differentiation, cell adhesion, cell apoptosis, and cell migration. *KRAS* is the most frequently mutated isoform in the *RAS* family, which constitutes 86% of *RAS* mutations (29). *KRAS* gene is a proto-oncogene that encodes a 21-kDa GTPase transducer protein, which has two states (30, 31): GTP-binding (active) and GDP-binding (inactive). In response to extracellular stimuli, the intracellular cycling between these two states is regulated by the guanine nucleotide exchange/releasing factors (GEFs/GRFs) and the GTPase activating proteins (GAPs) and further influence three major signaling pathways in both normal and transformed cell types, namely the mitogen-activated protein kinase (MAPK) pathway, phosphoinositide 3-kinase (PI3K) pathway, and the Ral-GEFs pathway, to promote cell survival, proliferation, and cytokine secretion (29, 32–34). The binding of GTP causes a conformational change of KRAS protein that involves two important regions (35): Switch Ⅰ (amino acids 30–38) and Switch Ⅱ (amino acids 59–67). The Switch regions Ⅰ and Ⅱ play pivotal roles in the binding of regulators and effectors. KRAS protein has a weak intrinsic GTPase activity [G domain of the KRAS protein (36)], which could be amplified by GAPs and facilitate the return of the GTP-bounding active form to its inactive GDP-bounding form (37–40). In contrast, GEFs/GRFs could catalyze the exchange of GDP for GTP.

Mutations in *KRAS* could impair the intrinsic GTPase activity of KRAS protein, and the protein becomes unresponsive to GAPs that maintain the KRAS protein in its active form (39, 41). The mutant KRAS protein causes aberrant and uncontrollable cell growth and cell transformation without being affected by upstream molecules in many cancer types. Different mutant KRAS proteins have unique biochemical behaviors in intrinsic/GAPs-stimulated GTPase activities and interactions with effectors (39, 42). Over 80% of *KRAS* mutations occur at codon 12 (43), including the commonly occurring glycine-to-cysteine mutation (KRAS G12C), the glycine-to-valine mutation (KRAS G12V), and the glycine-to-aspartic acid mutation (KRAS G12D), likely to produce a steric hindrance that prevents binding of GAPs and decreases GAP-stimulated GTPase activity. Different conformations produced by distinct mutant KRAS substitutions can lead to an altered association with downstream effector molecules, resulting in preferential signal transduction and biologic behavior that may impact clinical outcomes and response to therapy (43). In the previous studies (44, 45), KRAS G12C and KRAS G12V have been shown to preferentially signal through the Ral-GEFs pathway with worse PFS than other KRAS mutant or wild-type KRAS, whereas KRAS G12D has been shown to preferentially signal through the MAPK and the PI3K pathways. *KRAS* mutations drive the tumorigenesis of the three most lethal cancers (lung cancer, colorectal cancer, and pancreatic cancer) (46). Different mutations of KRAS have shown tissue-specific incidences, such as G12V/C in lung cancer (47, 48) and G12R in pancreatic cancer (49). Novel targeting therapies are currently being pursued as potential treatments for KRAS-mutant non-small-cell lung cancer (43, 50) and KRAS-mutant colorectal cancer (51, 52), which might be worthwhile for KRAS-mutant EC in the future.

In EC, the incidence of *KRAS* mutation is increasing globally (Table 1), which was reported to be approximately 15.0% in the past (37, 61–63). Ahmed et al. (64) found the presence of *KRAS* mutation in both complex mucinous changes and mucinous adenocarcinoma, indicating that *KRAS* mutational activation was implicated in the pathogenesis of a significant subset of endometrial mucinous carcinoma. Xiong et al. (65) noticed that the *KRAS* status in the neoplastic lesion presented on the surface of the endometrium was different from that in the benign metaplastic areas, while Van der Putten et al. (66) found that *KRAS* mutation appeared both in the hyperplastic lesions and the adjacent benign endometrial tissues. Other studies (46, 62) also supported that *KRAS* mutation occurred in the early stages of type I EC (endometrioid) before clonal expansion, which is similar to the role of *KRAS* mutation in colorectal cancer (67, 68). Endometrial atypical hyperplasia (EAH) is now believed to be the precancerous lesion of EEC, in which *KRAS* was also a highly mutated gene (63, 64, 69). Contrarily, some studies found no *KRAS* mutation in endometrial atypical hyperplasia (70, 71), probably due to the small sample size. On the other hand, Tsuda et al. (72) stressed the role of *KRAS* in predicting invasive proliferation of well-differentiated (grade 1) tumors. Birkeland et al. (73) noticed an increase in *KRAS* amplification and *KRAS* mRNA expression during the transition from primary to metastatic disease. All these data suggest that *KRAS* mutation may be crucial to the proliferation and invasion of EEC, and might be used as a predictive marker to predict a potential malignant transition and progression. It has been reported that *KRAS* mutation is associated with obesity (73, 74), a high-risk factor for the development of EEC (75), and decreased expression of estrogen receptor (76), suggesting a potential crosstalk between *KRAS*-mediated signaling pathway and the estrogen receptor activated signaling pathway. Recently, Ahmed et al. (64) demonstrated that *KRAS* mutation had a positive predictive value of 88% for complex atypical hyperplasia (the precancerous lesion of EEC) or adenocarcinoma. The *KRAS* mutation testing may be valuable for predicting the therapeutic effect of conservative treatment in EC, and the patients carrying specific mutations of *KRAS* may profit from the targeted therapy. However, the *KRAS* status of EC patients with conservative treatment has not been studied yet. Although current molecular classification shows potential predictive value in fertility sparing management, there are still proposed candidates for further risk stratification, such as *KRAS* (56, 77). If *KRAS* mutation tracking, combined with other biomarkers, could be completed during the follow-up of conservative treatment of EC patients, it may be possible to further understand the actual significance of *KRAS* mutation and help determine whether it can be an indicator of prognosis in conservative treatment. However, the *KRAS* status has not been reported during the follow-up of conservative treatment of EC/EAH patients. It is unclear whether *KRAS* status will change during follow-up, and how these changes will be associated with the effect of fertility preservation treatment.

**TABLE 1:** *KRAS* mutation frequencies in different EC/EEC studies.

Studies	*n* =	Sample	Mutation rate%	Relative mutation distribution (%) by condon				Relative mutation distribution (%) by nucleotide substitution					
				Codon 12	Codon 13	Codon 61	Other codons	G12D	G12V	G12C	G12A	G13D	Others
(53)	2293	EC	427 (18.6%)	87 (100%)	-	-	-	29 (33.3%)	25 (28.7%)	9 (10.3%)	8 (9.2%)	13 (14.9%)	3 (3.4%)
	1242	EEC	298 (24.0%)										
(54)	464	EEC	87 (18.8%)										
(55)	385	EC	64 (16.6%)										
	306	EEC	54 (17.6%)										
(56)	248	EC	53 (21.4%)	29 (70.7%)	12 (29.3%)	-	-	15 (36.6%)	7 (17.1%)	1 (2.4%)	5 (12.2%)	11 (26.8%)	2 (4.9%)
(57)	199	EC	45 (22.6%)										
(58)	197	EC	41 (20.8%)										
(59)	109	EC	23 (21.1%)										
	87	EEC	22 (25.3%)										
(60)	100	EC	23 (23.0%)										
	82	EEC	20 (24.4%)										

EEC, endometrioid endometrial cancer; EC, endometrial cancer.

As rates of obesity and metabolic syndrome have risen, rates of EC have also increased, creating demands of reproductive-aged patients for fertility preservation. It may be of clinical significance to use the molecular classification in the fertility-preserving EC/EAH patients to guide individualized treatment and prognostic evaluation in the future. Notably, detecting the *KRAS* status might help to further stratify the therapeutic response of fertility-preserving EC/EAH patients. Screening such patients with specific *KRAS* mutations might also help the introduction of the targeted therapy and the improvement of the fertility-sparing efficacy.

## Footnotes

1
https://www.nccn.org/professionals/physician_gls/default.aspx#uterine

## Abbreviations

EC, endometrial cancer; EEC, endometrioid endometrial cancer.
